# Characterization of Nrf1b, a Novel Isoform of the Nuclear Factor-Erythroid-2 Related Transcription Factor-1 That Activates Antioxidant Response Element-Regulated Genes

**DOI:** 10.1371/journal.pone.0048404

**Published:** 2012-10-29

**Authors:** Eric K. Kwong, Kyung-Mi Kim, Patrick J. Penalosa, Jefferson Y. Chan

**Affiliations:** Department of Laboratory Medicine and Pathology, University of California Irvine, Irvine, California, United States of America; North Carolina State University, United States of America

## Abstract

Nuclear factor E2-related factor 1 (Nrf1) is a basic leucine zipper transcription factor that plays an important role in the activation of cytoprotective genes through the antioxidant response elements. The previously characterized long isoform of Nrf1 (Nrf1a) is targeted to the endoplasmic reticulum and accumulates in the nucleus in response to activating signals. Here we characterized a novel Nrf1 protein isoform (Nrf1b) generated through an alternative promoter and first exon that lacks the ER targeting domain of Nrf1a. The 5′-flanking region of Nrf1b directed high levels of luciferase reporter expression in cells. RT-PCR and Western blotting showed Nrf1b is widely expressed in various cell lines and mouse tissues. Immunoblot analysis of subcellular fractions and imaging of green fluorescence protein (GFP)-tagged Nrf1b demonstrate Nrf1b is constitutively localized to the nucleus. Nrf1b can activate GAL4-dependent transcription when fused to the heterologous GAL4 DNA-binding domain. Gel-shift and coimmunoprecipitation experiments demonstrate that Nrf1b forms a complex with MafG, and expression of Nrf1b activates the expression of antioxidant response element containing reporters and genes in cells. These results suggest Nrf1b is targeted to the nucleus where it activates ARE-driven genes and may play a role in modulating antioxidant response elements.

## Introduction

Nuclear factor erythroid-derived 2-related factor 1 (Nrf1) is a member of the Cap ‘n’Collar (CNC) family of transcription factors that includes 3 closely related members, Nrf2, Nrf3, and p45NFE2 [1], [2], [3], [4]. These CNC members contain a conserved basic-leucine-zipper (bZIP) domain known to heterodimerize with small Maf oncoproteins (MafF, MafG, MafK) and bind to *cis*-acting sequences [5]. Nrf2 knock-out mice demonstrated the importance of Nrf2 in conferring protection from oxidative stress-related diseases in various organs, while Nrf1 proved to be vital in mouse embryonic development [6]. Nrf1 and Nrf2 regulate expression of antioxidant genes through the antioxidant response elements (AREs) [6], [7], [8]. The ARE is present in promoters of phase-2 detoxification enzymes and genes with cytoprotective function [9], [10], [11].

Despite the similar functions of Nrf1 and Nrf2 in maintaining cellular homeostasis and protecting against oxidative damage, Nrf1 has been suggested to play a distinct role in regulating ARE-driven genes. For instance, studies of liver-specific and brain-specific Nrf1 knockout mice have elucidated Nrf1's importance in preventing non-alcoholic steatohepatitis and neurodegeneration, respectively [12], [13]. In addition, metallothionein genes (MT1 and MT2) have been shown to be activated exclusively by Nrf1 [14]. Nrf1 has also been shown to play a key role in the coordinate regulation of proteasome genes [13], [15]. In addition to activating antioxidant genes, Nrf1 is involved in the regulation of developmental and cellular processes. A zinc finger transcription factor known as Osterix, involved in the differentiation of osteoblast and bone, formation has been identified as an Nrf1 target gene [16]. Studies also link Nrf1 as a modulator in the inflammatory response. Transforming growth factor-β (TGF-β) acts as a negative regulator of inducible form of nitric oxide synthase (iNOS), a regulator of the vascular response in inflammation and injury. Treatment of TGF-β in human smooth muscle cells led to an increased expression and binding of Nrf1 onto ARE-containing sites of the iNOS promoter [17]. Nrf1 has been reported to function as a negative regulator of odontoblast differentiation through interaction with CEBP-β to repress the dentin sialophosphoprotein (DSPP) gene that encodes for DSP and DPP in terminally differentiated odontoblasts [18].

The Nrf1 gene encodes several protein isoforms [19], [20], [21]. The 120 kDa isoform of Nrf1 has been shown to be targeted to the endoplasmic reticulum (ER) by an N-terminal transmembrane domain [22], [23], while the shorter 65 kDa isoform is found in the nuclear compartment [21]. The mechanism by which p120Nrf1 is targeted to the nucleus is not known but may involve retrotranslocation from the ER and/or proteolytic cleavage of the transmembrane domain during ER stress [22], [24], [25]. The p120Nrf1 isoform has been shown to be upregulated in oxidative stress [26] and shares structural similarities with Nrf2 including the Neh2 domain that serves to target Nrf2 for ubiquitination and degradation through interaction with the Keap1 protein [22]. Although Keap1 interacts with p120Nrf1, Keap1 does not seem to regulate p120Nrf1 function [22], [23]. Recent studies have shown p120Nrf1 to be negatively regulated by the components of the SCF (Skp1-Cul1-Fbox protein) ubiquitin ligase for degradation via the ubiquitin-proteasome pathway. The F-box Fbw7 protein has been shown to directly bind p120Nrf1 and promote its ubiquitination and degradation [27]. In addition to Fbw7, p120Nrf1 degradation is also promoted by another F-box protein β-TrCP [28]. The p65Nrf1 is an N-terminally truncated Nrf1 protein that is presumably generated by alternative translation from in-frame start codons downstream from the normal start codon present in the Nrf1 transcript. The function of p65Nrf1 in gene regulation is unclear, but evidence suggests that the p65Nrf1 antagonizes ARE-mediated gene transcription by inhibiting Nrf2 function [21].

Through alternative promoters and mRNA processing, multiple transcripts are generated from a single gene to encode for proteins with different properties and functions. It has been predicted that more than a third of human genes use alternative splicing to generate mRNA variants derived from one gene [29]. In conjunction with alternative splicing, usage of alternative transcription start sites provide a mechanism for generating tissue specific protein isoforms, as well as a mechanism for regulating levels of expression, and generating mRNA isoforms with different stability and translation efficiency [30]. In this report, we characterized Nrf1b, a novel isoform of Nrf1 with an apparent mass of 95 kDa that is distinct from the p120Nrf1 and p65Nrf1. Nrf1b contains a unique N-terminus encoded by an alternative first exon, and lacks the N-terminal ER targeting sequence of p120Nrf1 (herein termed Nrf1a). While both Nrf1b and p65Nrf1 are truncated isoforms of Nrf1 and are localized in the nucleus, Nrf1b interacts with MafG to activate ARE-containing genes. Overall, these results suggest that Nrf1b, analogously to Nrf1a, can activate ARE-driven genes to induce stress response in cells.

## Materials and Methods

### Reagents

Tissue culture media, fetal calf serum, and V5-Tag mouse monoclonal antibody were purchased from Invitrogen (Carlsbad, CA). MafG rabbit polyclonal antibody was purchased from GeneTex (Irvine, CA). Rabbit polyclonal antibody generated against Nrf1-glutathione transferase fusion protein was previously described [22]. Phusion Site-Directed Mutagenesis Kit was purchased from New England Biolabs (Beverly, MA). BioT transfection reagent was purchased from Bioland Scientific (Paramount, CA). Subcellular Protein Fractionation Kit was purchased from Thermo Scientific (Rockford, IL). PCR primers and oligonucleotides were from Sigma-Aldrich. Horseradish peroxidase-linked anti-rabbit IgG and anti-mouse IgG were from Cell Signaling (Beverly, MA). Chemiluminescent detection system for immunoblot (ECL), and protease inhibitor mix were purchased from Pierce Biotechnology (Rockford, IL). Restriction enzymes, TaqDNA polymerases, and other modification enzymes were purchased from New England Biolabs (Beverly, MA). Lightshift Chemiluminescent EMSA Kit was purchased from Thermo Scientific (Rockford, IL).

### Plasmids

The V5-tagged expression vector encoding Nrf1a (pEF1Nrf1a-V5) was generated by PCR amplification of the Nrf1 cDNA using GGAATTCTTCAGCAATGCTTTCTCTG, and CCGCGGCCGCTTTCTCCGGTCCTTTC primers, and cloned into the EcoR1 and NotI sites of pEF1-V5His (Invitrogen, Carlsbad, CA). Nrf1b-V5 construct was generated by PCR amplification using GACATAGATCTGATTGACATCCTTTG, TGTCGACCGAATTCCACCACACTG primers and Nrf1a as template DNA. The subsequent PCR product was then used to add the unique 36nt region encoding the 5′ of Nrf1b by another round of PCR amplification using the forward-CTCACTGCAGCCTCTGCGGACATAGATCTGATTGACATCCTTTG and reverse- ATGGGACTCCCACCCCATTGTCGACCGAATTCCACCACACTG primers, and then cloned into EcoR1 and NotI sites of pEF1-V5His plasmid. The Nrf1b-Luciferase construct was generated by PCR amplification of the mouse genomic DNA sequence using CACTGCTAGCGCCGGTATTAT, AGAAGCTCGAGTGAGTCGGGA primers that spans from -1021nt to +23nt of the Nrf1b open reading frame, and cloned into the NheI and XhoI sites of the pGL3-basic vector. The 3xARE-Luciferase construct containing three ARE (indicated in upper case) was obtained by annealing the complementary oligos, ctagccgtgggcacga TGACTCTGCA ccgcctcctctgagccgtgggcacga TGACTCTGCA ccgcctcctctgagccgtgggcacga TGACTCTGCA ccgcctcctctgc and tcgagcagaggaggcgg TGCAGAGTCA acgtgcccacggctcagaggaggcgg TGCAGAGTCA tcgtgcccacggctcagaggaggcgg TGCAGAGTCA tcgtgcccacgg, and cloned into the NheI and XhoI sites of the pGL3-promoter vector. The Nrf1bEGFP construct was generated by PCR amplification of Nrf1b using primers GGTGGAATTCGGTCGACAAT and TCTAGACTCGACCGGTCGCTTT and cloned in-frame into the EcoRI and AgeI sites of pEGFP-N1 vector (Clontech, Palo Alto, CA). Nrf1 constructs fused with the Gal4 DNA-binding domain were generated by PCR amplification of Nrf1 and subcloned into the KpnI and SacI sites of the pSG424 vector. Nrf1a-Gal4 was amplified using AATTCGGTACCCAATGCTTTCTCT and TCGGATGGAGCTCAGCTG primers and Nrf1a-V5 as template DNA. Nrf1b-Gal4 was amplified using AATTCGGTACCCAATGGGGT and TCGGATGGAGCTCAGCTG primers and Nrf1b-V5 as template DNA. The GCLM-Luciferase reporter was previously described.

### Transient Transfection

HEK293T and COS cells were grown in Dulbecco's modified Eagle's medium supplemented with 10% fetal calf serum, 100 μg/ml of each streptomycin, and 100 units/ml penicillin at 37°C in a humidified, 5% CO_2_ atmosphere. Hepa1c1c7 cells were grown in alpha minimum essential medium supplemented with 10% fetal calf serum, 100 μg/ml of each streptomycin, and 100 units/ml penicillin at 37°C in a humidified, 5% CO_2_ atmosphere. Cells were transfected using BioT reagent according to the manufacturer's protocol. The cells were plated at least 12 h before transfection. The cells were harvested 48 h after transfection and cellular extracts were prepared.

### Tissue sample collection

Adult C57BL/6 mice at 8–12 weeks of age were euthanized by cervical dislocation and various tissues were collected on ice and stored at −80°C until processing for mRNA and protein studies. The animal study protocol was reviewed and approved by our institution's Animal Care and Use Committee.

### Immunoblotting

Cells were lysed in cold RIPA buffer (50 mM Tris-HCl pH 7.4, 150 mM NaCl, 1% Triton X-100, 1% sodium deoxycholate, 0.1% SDS, 1X Protease Inhibitor) and centrifuged for 15 min at 4°C. Tissue samples were homogenized in cold RIPA buffer using a polytron homogenizer. Protein concentrations were determined using the Bio-Rad Protein Assay reagent and Bradford protein assay. An equal volume of 2 X SDS sample buffer (100 mM Tris, pH 6.8, 25% glycerol, 2% SDS, 0.01% bromphenol blue, 10% 2-mercaptoethanol) was added to cell lysates, and the mixture was boiled for 5 min. Samples were resolved by SDS-PAGE and transferred to nitrocellulose membranes. After blocking with 5% skim milk in TBS-T (150 mM NaCl, 50 mM Tris-HCl, pH 8.0, and 0.05% Tween 20), the membranes were probed with the indicated primary antibodies overnight at 4°C followed by a incubation with a horseradish peroxidase-conjugated secondary antibody. The antibody-antigen complexes were detected using the ECL system.

### RNA Isolation and RT-PCR

Total RNA was extracted using UltraSpec RNA (Biotecx). cDNA was synthesized from 10 μg total RNA in 20-μL reactions containing 1 × RT buffer, 1 mM dNTPs, 0.3 μg random hexamer, 40 U of RNase inhibitor, and 250 U of Moloney murine leukemia virus reverse transcriptase. Reverse transcription reactions were incubated at 72°C for 5 min, 25°C for 10 min, and followed by 42°C for 60 min. Nrf1b cDNA transcripts were amplified by PCR with cycling conditions consisting of 95°C for 5 min and 35 cycles of 95°C for 30 s, 60°C for 30 s, and 72°C for 30 s. Expression levels were normalized to 18s levels. Quantitative RT-PCR was performed by amplifying cDNA in a Step One Plus PCR machine (Applied Biosystems) using FastStart SyBr Green reagent (Roche) in duplicates in 10-μL reaction volumes. PCR cycling conditions consist of 95°C for 15 min and 45 cycles of 95°C for 30 s, 60°C for 30 s, and 68°C for 45 s. Expression levels were calculated relative to GusB levels as endogenous controls.

### Primer extension analysis

A FAM-labeled complementary primer (5′- GTGAGTCGGGACTCCCACC -3′) to the region from +5 to +23 of the mouse Nrf1b mRNA was hybridized with PolyA mRNAs from MEF cells, and extended using AMV reverse transcriptase. Extended products were resolved by capillary electrophoresis on an ABI machine. The sequence ladder was generated from the same primer using a template plasmid DNA containing upstream promoter region of the mouse Nrf1b gene.

### Immunocytochemistry and Microscopy

COS cells were grown on coverslips and Nrf1b-EGFP was transfected into cells using BioT. One day after transfection, the coverslips with cells were washed with PBS and fixed with 4% paraformaldehyde for 15 min, followed by three washes with PBS. The coverslips were then mounted on slides and visualized with a Nikon epifluorescent microscope.

### Subcellular Fractionation

Subconfluent HEK293 cells were transfected with Nrf1b-V5 expression vector in a 60-mm dish. After 48 h, cells were washed with cold phosphate-buffered saline and collected by centrifugation. Subcellular fractions were obtained using Thermo Scientific Subcellular Protein Fractionation Kit following manufacturer's protocol. Equal proportions of each fraction were resolved with SDS-PAGE followed by immunoblot analysis with indicated antibodies.

### Co-Immunoprecipitation

Subconfluent HEK293 cells were transfected with Nrf1b-V5 expression vector and were lysed in radioimmune precipitation assay buffer 48 h after transfection. The lysates were cleared by centrifugation for 15 min at 4°C followed by pre-clearing protein samples with protein-G Sepharose beads by a 1 h incubation in the cold. The protein samples were incubated with anti-V5 antibody or no antibody as a control overnight in the cold. The next day, protein-G Sepharose beads were added followed by 1 hour incubation in the cold. The beads were collected by brief centrifugation and then washed extensively with radioimmune precipitation assay buffer. The proteins were eluted in 1 X SDS sample buffer and heated at 95°C for 5 min. The samples were separated by SDS-PAGE and transferred onto a nitrocellulose membrane, followed by immunoblotting with indicated primary antibodies and horseradish peroxidase-conjugated secondary antibodies. Detection of peroxidase signal was performed using the chemiluminescence method.

### Gel Shift Assay

Nrf1b and MafG were synthesized using the rabbit reticulocyte TNT system from Promega. Binding reactions were carried out in 10 mM Tris pH 7.5, 50 mM KCl, 1 mM dithiothreitol, 1 μg of poly(dI/dC), 4% glycerol containing biotin-labeled double-stranded oligonucleotide probes corresponding to the antioxidant response element of human NQO1 gene promoter (5′-CAGTCACAGTGACTCAGCAGAATCT-3′). Mixtures were incubated at room temperature for 20 min, and the DNA-protein complexes were resolved on nondenaturing 4% acrylamide gels in 0.5× TBE (45 mM Tris borate, 1 mM EDTA) at 4°C temperature. Electrophoretic transfer of binding reactions to nylon membrane and detection of biotin-labeled DNA were performed using Lightshift Chemiluminescent EMSA Kit following manufacturer's protocol. For supershift experiments, indicated antibodies were added 10 min prior to the addition of ARE probe to the binding reaction mix.

### Luciferase Assays

HEK293 cells were transfected in 24-well plates using BioT following manufacturer's protocol. After 24 h, cellular extracts were prepared and luciferase activities were measured with the Dual Luciferase Reporter Assay kit (Promega) using a TD-20/20 luminometer (Turner Designs). Firefly luciferase values were normalized to *Renilla* luciferase control. Transfection experiments were repeated at least three times and data points were calculated as the mean of the results (3 wells/experiment) +/− S.D.

### Statistical analysis

Data are expressed as means ± SEM, and statistical analysis using Student's t-test was done with Microsoft Excel (Redmond, WA). * indicates p values <0.05, and considered significant.

## Results

### Nrf1b encodes an Nrf1 Isoform with a novel N-terminus

**Figure 1:**
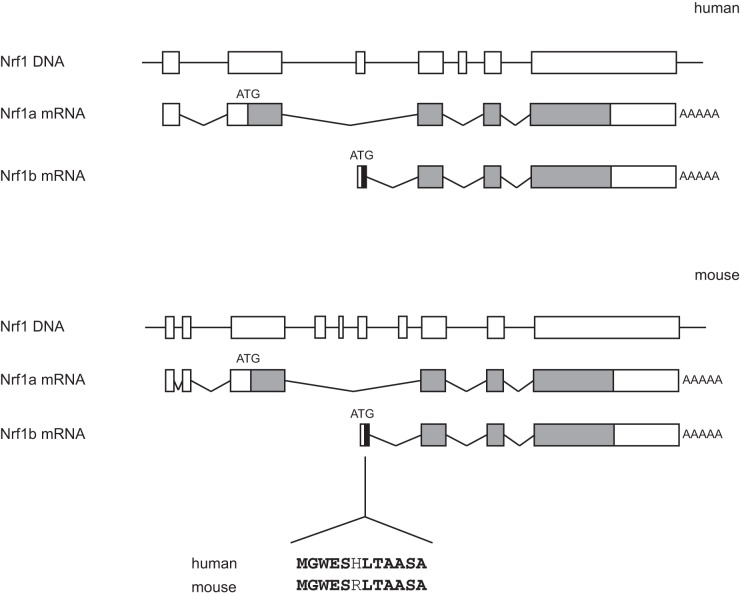
Nrf1b encodes a novel Nrf1 protein. Schematic diagram of human and mouse Nrf1 genomic sequences, and depiction of Nrf1a and Nrf1b transcripts. Solid and open boxes represent coding regions and untranslated regions, respectively. Solid lines represent introns and 5′-flanking regions.

Alternative promoter usage and splicing are important mechanisms in generating protein diversity. Human and mouse sequence data in the Ensembl Genome Browser showed a previously uncharacterized Nrf1 transcript with a distinct first exon. We propose to name this new isoform Nrf1b, and the previously characterized p120Nrf1 as Nrf1a. The first exon of Nrf1b is located between the first and second exon of Nrf1a and codes for 12 amino acid residues that are identical in both human and mouse except for the histidine to arginine substitution at position 6 in the mouse sequence (Fig. 1). The mouse Nrf1b cDNA is 3547 nucleotides in length, and encodes a protein of 583 amino acid residues that is identical to Nrf1a up to residue 13 (Fig. 1). Nrf1b lacks the N-terminal ER targeting sequence of Nrf1a, suggesting that Nrf1b might be a constitutively nuclear protein.

### Genomic sequence upstream of Nrf1b has transcription promoting activity

**Figure 2:**
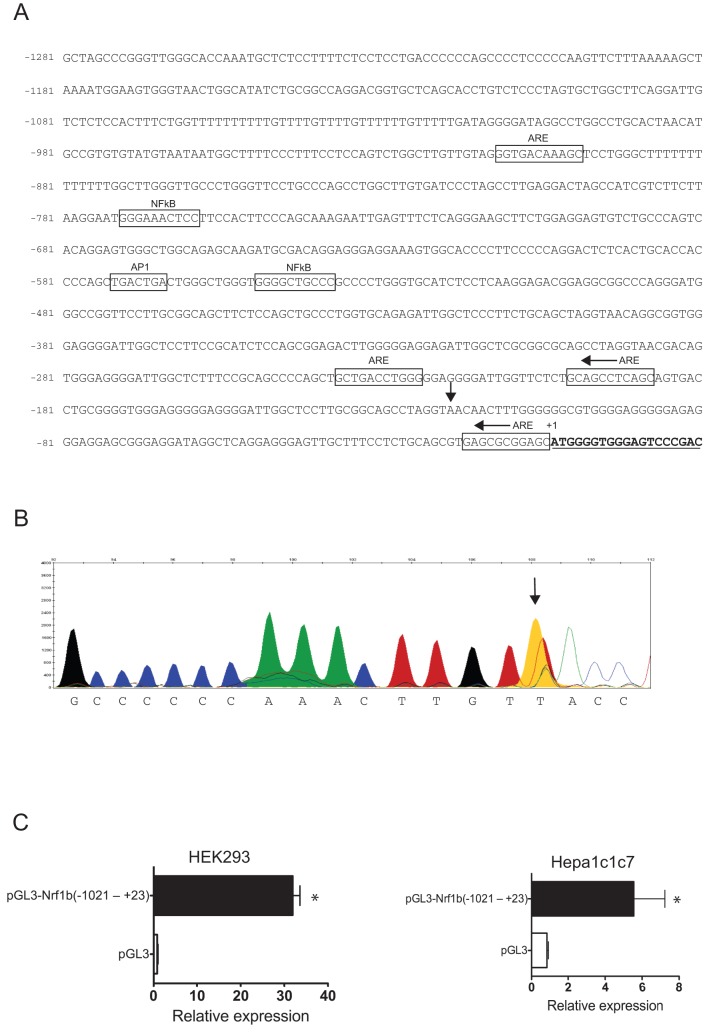
Nrf1b is derived by alternative promoter usage. (**A**)**.** Nucleotide sequence and identification of putative cis-acting elements in the 5′-flanking region of Nrf1b exon 1. Numbering is relative to the first nucleotide of the initiation codon (ATG) designated as +1. Vertical arrow represents the transcription start site identified by primer extension analysis, and coding region is underlined. The cis-acting elements containing consensus sequences are boxed. (**B**)**.** Primer extension result with NIH3T3 mRNA. The peak corresponding to a 108-bp elongation product (FAM-labeled cDNA) is indicated by an arrow. Labeled cDNA was aligned with the sequence electropherogram to identify the base at which transcription starts for Nrf1b. Nucleotides are indicated on the x-axis. (**C**)**.** Relative luciferase activities of the mouse Nrf1b promoter construct in HEK293 and Hepa1c1c7 cells. Negative control consisted of the pGL3-Basic control. Luciferase activities were normalized to *Renilla* luciferase from pRL-TK. Histograms show mean ± SD of three independent experiments with triplicate samples per experiment. *P<0.05.

To determine whether the Nrf1b transcript is produced from an alternative promoter, primer extension analysis was performed. Using a primer hybridizing within exon 1 of Nrf1b, a major extension product was detected in RNA from NIH3T3 cells, placing the transcription initiation site 93bp upstream of the ATG codon (Fig. 2A arrowhead, 2B). Sequence inspection of the 1.5-kb gene segment upstream of ATG encoding Nrf1b did not reveal an apparent TATA box motif. However, canonical cis-acting binding motifs were found including several antioxidant response elements and a consensus sequence for AP1 and NFKB sites (Fig. 2B). To investigate whether the genomic sequence upstream of Nrf1b has transcription promoting activity, the region located −1021 to +23 nucleotides relative to the initiation ATG site of Nrf1b was cloned into the pGL3-Luciferase vector. Nrf1b-Luciferase reporter plasmids were transiently transfected into HEK293 and Hepa1c1c7 cells, and promoter activity determined by measuring firefly luciferase activity normalized to Renilla luciferase activity. Reporter expression in cells transfected with Nrf1b-Luciferase plasmid were markedly higher compared to cells expressing the pGL3-Luciferase control vector (Fig. 2C). This finding is consistent with the idea that the intron promoter drives transcription of the Nrf1b transcript in the mouse.

### Nrf1b is widely distributed

**Figure 3:**
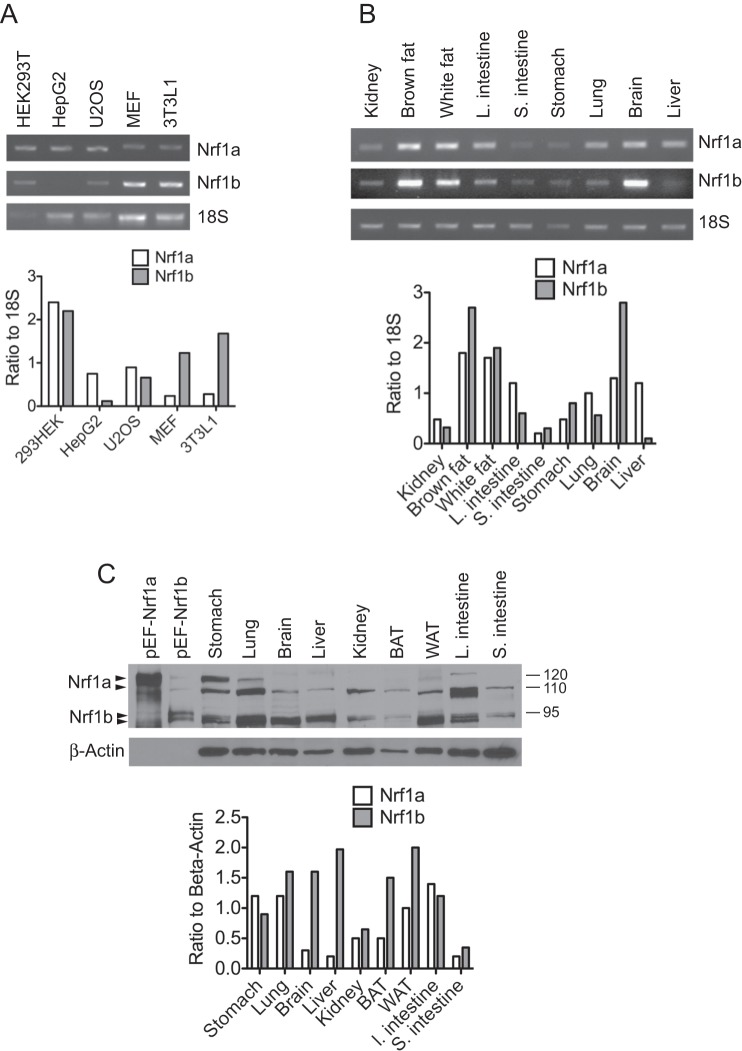
Nrf1b expression is widely distributed. Nrf1a and Nrf1b mRNA expression patterns were analyzed by RT-PCR in various cell lines (**A**) and mouse tissues (**B**)**.** Nrf1a and Nrf1b cDNA was amplified by PCR for 30 cycles and 18S was amplified for 20 cycles. Histograms show relative Nrf1a and Nrf1b expression normalized against 18S. (**C**)**.** Western blot of different mouse tissues probed with Nrf1 antibody. HEK293 cells transfected with pEF1-Nrf1a (lane 1), and pEF1-Nrf1b (lane 2) were used as controls for detection of the Nrf1a and Nrf1b isoforms by the Nrf1 antibody. Beta-actin was used as a loading control.

To determine the expression pattern of Nrf1b and compare it to Nrf1a, RT-PCR analysis was done using isoform-specific primers. Nrf1a and Nrf1b transcripts were detected in different mouse and human cell lines (Fig. 3A). Both transcripts were also present in various mouse tissues examined (Fig. 3B). Compared to Nrf1a, Nrf1b levels were elevated in brown fat and brain, but were reduced in liver. To confirm the RT-PCR data, protein distribution of Nrf1a and Nrf1b was examined. Western blot analysis using a polyclonal antibody against Nrf1 was done to detect endogenous Nrf1 proteins, and transiently expressed V5-epitope tagged Nrf1a and Nrf1b isoforms were used as controls. Consistent with previous data, Nrf1a is separated into two major bands, with the upper band at 120 kDa representing the ER membrane bound form of Nrf1a, and a lower band migrating as 110 kDa representing free form of Nrf1a [22]. Both endogenous and transiently expressed Nrf1b migrated at an apparent molecular weight of approximately 95 kDa (Fig. 3C). In addition, we noted that Nrf1b migrated as a doublet suggesting that it is post-translationally modified. As shown in figure 3C, both protein isoforms were widely distributed. Increased levels of Nrf1b protein was detected in brain and brown fat. In contrast to findings by RT-PCR, lung, liver and white fat showed increased Nrf1b compared to Nrf1a. The basis of this discrepancy between mRNA and protein levels is not known. From these results however, we conclude that Nrf1b is widely distributed with certain tissues containing higher levels.

### Nrf1b is detected in the cytoplasm and nucleus

**Figure 4:**
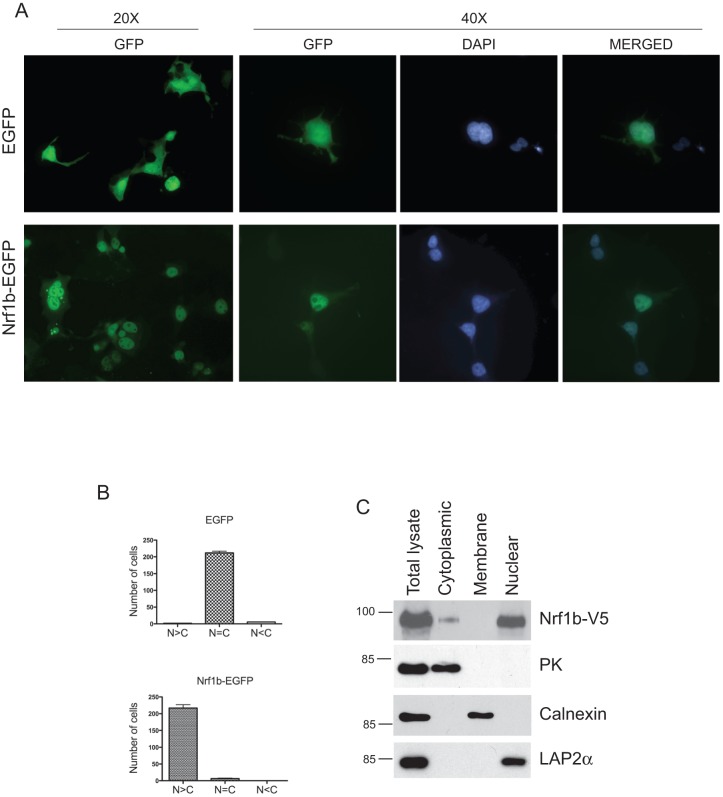
Nrf1b protein is localized in the nucleus. (**A**)**.** Epifluorescent micrographs showing COS7 cells transfected with Nrf1b-EGFP. (**B**) Quantitative analysis of the results in (B). The subcellular localization of EGFP and Nrf1b-EGFP was scored as follows: N>C, predominantly nuclear; N  =  C, evenly distributed between the nucleus and cytoplasm; N<C, predominantly cytoplasmic. Error bars represent the means ± SD of two independent experiments. (**C**)**.** Distribution of Nrf1b-V5 in cells. V5-tagged Nrf1b was harvested from HEK293 cells 48hr after transfection as described in the methods section, and analyzed by Western blot. The antibodies used for Western blotting are indicated on the right. Pyruvate kinase was used as a cytoplasmic marker, calnexin as an ER membrane marker, and lamina-associated polypeptide 2α (LAP2α) as a nuclear marker.

To determine the localization of Nrf1b, we expressed Nrf1b fused with EGFP in COS cells and visualized the cells by microscopy. As shown in Fig. 4A and B, fluorescence was uniformly distributed in control cells expressing EGFP. In contrast, fluorescence in cells expressing Nrf1b-EGFP fusion protein was mostly in the nucleus. To corroborate these results, Western blotting was performed on subcellular fractions of HEK293 cells expressing V5-tagged Nrf1b. Consistent with localization of Nrf1b-EGFP in COS cells, Nrf1b-V5 was detected in the nuclear fraction of HEK293 cells (Fig. 4C). In addition to the nucleus, Nrf1b-V5 was also detected in the cytoplasmic fraction of transfected HEK293 cells. However, Nrf1b-V5 was not detected in the membrane fraction, which underscores the importance of the N-terminal targeting sequence of Nrf1a in ER localization.

### Nrf1b is an activator of gene transcription

**Figure 5:**
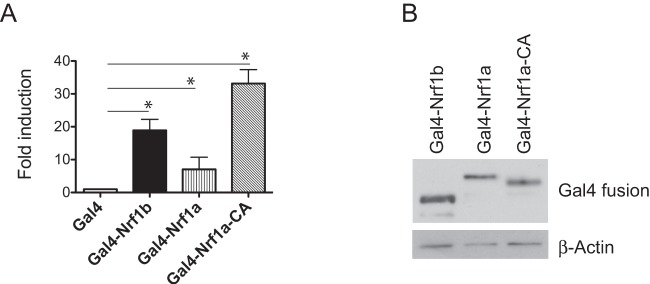
Nrf1b activates transcription when tethered to a promoter. (**A**) HEK293 cells were transfected with Gal4-Nrf1b, Gal4-Nrf1a or Gal4-Nrf1CA and a luciferase reporter containing five GAL4 binding sites. Cell extracts were prepared 24 h after transfection and the luciferase activities were normalized to *Renilla* luciferase from pRL-TK. Results are expressed relative to luciferase activities observed with vector alone. The bars depict the means ± SD of three independent experiments carried out in triplicates. *P<0.05 (**B**) Western blot analysis of cell extracts containing Gal4 DBD alone or Gal4 fusion constructs. Gal4 fusion constructs were transfected into HEK293 cells, and extracts were Western blotted for Gal4-fusion protein expression.

To assess the trans-acting potential of Nrf1b, a GAL4-based heterologous activation assay was performed. Nrf1b was fused in-frame with the DNA binding domain of Gal4 (AA 1–147), and the resulting Gal4-Nrf1b fusion expression plasmid was co-transfected into HEK293 cells with a luciferase reporter construct driven by GAL4 sites. Compared to control (Gal4DBD), the Gal4-Nrf1b vector activated luciferase expression by 20-fold in HEK293 cells (Fig. 5A), indicating that Nrf1b confers activation when recruited to a promoter. Gal4-Nrf1b is also more active than Gal4-Nrf1a (ER localized isoform of Nrf1). Gal4-Nrf1b was less active than the Gal4-Nrf1CA where the N-terminal membrane targeting sequence of Nrf1a has been deleted suggesting that ER liberated form of this protein is active. Western analysis confirmed that all Gal4 fusion proteins exhibited equivalent expression levels (Fig. 5B). These results indicate that Nrf1b-mediated gene activation represent an intrinsic property of the protein.

### Nrf1b interacts with MafG to bind the ARE

**Figure 6:**
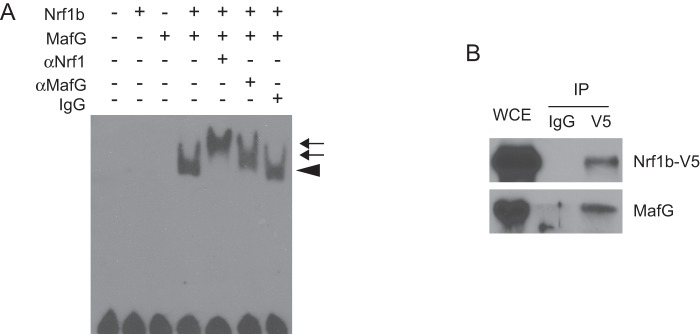
Nrf1b-MafG complex binds the ARE. (**A**) EMSA studies were performed with Nrf1b and MafG proteins generated by in vitro transcription and translation, and biotinylated DNA probe containing a consensus antioxidant response element described in Materials and Methods. Rabbit anti-Nrf1, anti-MafG, and IgG were used for super-shifts. Chevrons and arrows indicate shifted and super-shifted bands, respectively. (**B**) HEK293 cells transfected with Nrf1b-V5 were harvested 48 h after, and lysates were immunoprecipitated with anti-V5 antibody. Immunoprecipitates were then subjected to immunoblotting with anti-V5 or anti-MafG antibodies.

CNC-bZIP factors form heterodimers with other bZIP proteins, including small Maf proteins, to activate ARE-driven genes. To explore this possibility, we performed gel-shift assays using an ARE-containing oligonucleotide probe. No DNA-protein complex was detected when the ARE-probe was incubated with lysate, or lysate programmed with Nrf1b or MafG expression constructs (Fig. 6A). However, incubation of the ARE-probe with Nrf1b and MafG programmed lysates produced a prominent slower migrating band (Fig. 6A). This band was super-shifted by anti-Nrf1 and anti-MafG antibodies, but not by an unrelated control antibody indicating that both Nrf1b and MafG are present in the complex.

To assess interaction between MafG and Nrf1b in vivo, co-immunoprecipitation experiments were performed. Cell lysates prepared from HEK293 cells transiently transfected with V5-tagged Nrf1b expression plasmid were immunoprecipitated with anti-V5 antisera followed by immunoblot analysis. MafG was found in the immunoprecipitates from cells transfected with Nrf1b-V5 (Fig. 6B). This data indicates that Nrf1b can physically interact with MafG in vivo.

### Nrf1b activates ARE-driven genes

**Figure 7:**
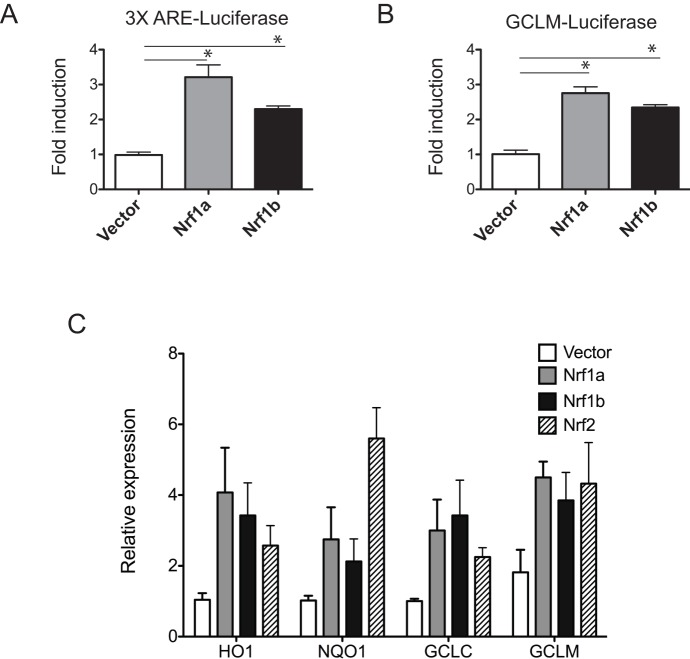
Nrf1b activates ARE-driven genes. Luciferase reporter bearing 3 copies of ARE (**A**)**,** or GCLM-luciferase reporter (**B**) was co-transfected with either Nrf1a or Nrf1b expression plasmid. Luciferase activities were normalized to *Renilla* luciferase from pRL-TK. Results are expressed relative to luciferase activities observed with vector alone. Histograms show the means of three separate experiments ± SD carried out in triplicates. *P<0.05 (**C**) Induction of endogenous ARE target genes. NIH3T3 cells were transfected with the indicated plasmids, and mRNA was harvested and analyzed 2 days after transfection by qRT-PCR. Histograms show mean ± SD (n = 4).

To determine whether Nrf1b can regulate ARE-mediated gene expression, Hepa1c1c7 cells were co-transfected with a luciferase reporter containing three copies of ARE and Nrf1b expression vector. As shown in figure 7A, expression of Nrf1b induced a 2-fold increase in promoter activity. In comparison, luciferase expression was induced 3-fold by Nrf1a. To substantiate the ability of Nrf1b to activate ARE-mediated gene expression, Hepa1c1c7 cells were co-transfected with Nrf1b and a luciferase reporter construct derived from the human GCLM proximal promoter that contains a functional ARE. Similar to the synthetic reporter, Nrf1b activated the GCLM reporter in Hepa1c1c7 cells (Fig. 7B). Next, we examined the ability of Nrf1b to stimulate endogenous ARE-bearing target genes in cells. NIH3T3 cells were transfected with Nrf1b, Nrf1a, Nrf2 or empty vector, and mRNA levels of various ARE-containing genes were determined using qRT-PCR. Enforced expression of Nrf1b, or Nrf1a, enhanced the levels of NQO1, HO1, GCLC and GCLM mRNA (Fig. 7C). As expected from previous studies, Nrf2 stimulated expression of ARE-bearing genes such as NQO1, HO1, GCLC and GCLM (Fig. 7C). These results indicate that Nrf1b can stimulate expression of ARE-containing genes.

## Discussion

Nrf1 is a member of the CNC basic leucine zipper family of transcription factors that play important roles in gene regulation and cellular development. Previous studies have shown Nrf1 as an important regulator of oxidative stress genes through interaction with the antioxidant response element. In this study, we have described a new isoform of Nrf1, which we call Nrf1b. Nrf1b transcript is derived from an alternative promoter site in the Nrf1 gene and encodes a structurally different protein than Nrf1a. In addition, we identified the presence of a functional promoter located upstream from the Nrf1b transcription start site. Endogenous protein expression of Nrf1b was observed in various tissues and cell lines. Furthermore, we have shown that Nrf1b is a positive transcription activator, and demonstrated that Nrf1b can activate endogenous ARE genes and may play a role in oxidative stress.

The Nrf1b transcript is detected in a wide range of tissues and cells, with higher levels present in certain tissues. Currently, it is not known if factors such as message stability play a role in tissue-specific differences in Nrf1b expression. However, alternative promoters can be used to exert different tissue or developmental specific patterns of expression. Large-scale genomic studies using ChIP-chip and analysis of 5′ cDNA ends suggest that a large proportion of mammalian genes involved in development and transcriptional control contain multiple promoters [31]. Examples of tissue-specific expression of protein isoforms via alternative promoters include the tumor suppressor protein 4.1B that produces two variable N-terminal kidney and brain 4.1B isoforms arising from two transcriptional promoters [32]. Similarly, the human HOX-5.1 gene encodes multiple transcripts that are differentially expressed in a tissue-specific manner during embryogenesis [33]. Therefore, the Nrf1b promoter identified here could mediate tissue-specific enrichment of Nrf1b. Although increased Nrf1b transcript was detected in brown fat, white fat and brain in comparison to other tissues, protein expression levels do not correlate with the mRNA levels. Elevated Nrf1b protein levels were also seen in lung, liver, and large intestine. The discrepancy between mRNA and protein expression suggests the possibility that Nrf1b is also regulated post-translationally. Recent studies have shown Nrf1a is an unstable protein that is regulated by the ubiquitin proteasome pathway [27]–[28]. Hence, it is possible that Nrf1b protein is also regulated by the UPS, which may contribute to the different protein expression levels observed. Whether Nrf1b is preferentially expressed over Nrf1a in certain tissue types under different environmental stressors remains to be determined. Nrf1b tissue specification and abundance may potentially contribute to unique functions not directly regulated by Nrf1a.

The transcription start site of Nrf1b is located 93 nucleotides upstream from the 5′ end of the Nrf1b cDNA. Transient transfection experiments showed that luciferase reporter plasmid containing 1-kb of 5′ flanking region of Nrf1b was active in both mouse and human cells. Nucleotide sequence analysis did not reveal consensus transcriptional regulatory sequences, such as TATA and CCAAT elements in the 5′ flanking region. Instead, the region is GC rich, which is found primarily in promoters of housekeeping genes [34]. Currently, the mechanism regulating Nrf1b expression via its promoter is not known. However, it is interesting to note that the putative promoter of Nrf1b contains several AREs. This raises the possibility of an autoregulatory positive loop to help sustain Nrf1b expression during cellular stress. An example of auto-regulation in transcription factors includes the closely related CNC-bZIP transcription factor Nrf2. The Nrf2 promoter contains ARE sites that are regulated by Nrf2 [35]. An attractive possibility is that Nrf1b may be self-regulated or regulated in part by Nrf1a or Nrf2 in response to oxidative-related stressors. The potential for regulation by Nrf2 and other CNC-bZIP factors would add another dimension to stress response. In addition to ARE sites, AP-1 and NFKB sites were also found in the 5′-flanking region of Nrf1b. Hence, it is possible that Nrf1b expression may be up-regulated in response to inflammatory stimuli via the NFKB signaling pathway. Further experimentation is required to elucidate these possibilities.

In addition to providing the capacity for differential regulation, alternative promoters generate protein diversity. Alternative promoters often generate different 5′-untranslated (UTR) regions or open reading frames that lead to the production of distinct protein products that contribute to functional diversification of proteins [36]. For example, the progesterone receptor (PR) gene encodes two functionally distinct isoforms termed PR-A and PR-B derived from distinct promoters, and each isoform regulates a different set of progesterone-dependent target genes [37]. Similarly, Nrf1b and Nrf1a are identical except that the first two exons encoding 174 amino acids at the N-terminus of Nrf1a are replaced by a new exon in Nrf1b encoding a novel N-terminus made up of 12 amino acids in length. The substitution results in the absence of the Nrf1a endoplasmic reticulum targeting domain preventing sequestration to the ER while promoting localization to the cytoplasm and nucleus. In support of this, our data demonstrates that Nrf1b protein is enriched in the nucleus and cytoplasm. The distinction from Nrf1a also suggests that Nrf1b may modulate expression of genes constitutively. Alternatively, the expression and intracellular trafficking of Nrf1b may be regulated by KEAP1. It was previously shown that Nrf1a shares structural similarities with Nrf2, which includes the Neh2 (Nrf2-ECH homology 2) domain that serves to destabilize Nrf2 through interaction with the KEAP1 protein. Although Nrf1a interacts with KEAP1, the location and function of Nrf1a is not regulated by KEAP1 [22]. Since Nrf1b possesses the Neh2 domain, it is reasonable to speculate that the cellular localization and stability of Nrf1b may be regulated by KEAP1. Further investigation is needed to determine these possibilities.

EMSA and co-immunoprecipitation data indicate that Nrf1b interacts with MafG and binds ARE. This is expected as both Nrf1a and Nrf1b share the same basic leucine zipper domain. Furthermore, our transfection data demonstrates that Nrf1b isoform acts as a transcriptional activator. Nrf1b functioned as an activator when fused with the GAL4 DBD. Previous studies showed that the deletion of the N-terminal ER signal sequence of Nrf1a (referred to as Nrf1CA) resulted in a constitutively nuclear localization of the protein that was more active than wild type Nrf1a in ARE gene transcription [22]. Nrf1b is similar to Nrf1CA in that it is missing the N-terminal ER targeting signal. In this regard, Gal4-Nrf1b was also more active than Gal4-Nrf1a fusion protein, but was less active compared to Gal4-Nrf1CA, which is consistent with the idea that Nrf1a requires processing in order for it to be released from the ER membrane. Additionally, Nrf1b can stimulate activation of a synthetic promoter containing ARE sites, as well as the human GCLM promoter. The potential role of Nrf1b in modulating ARE function was further supported by our finding that enforced expression of Nrf1b induced various ARE-bearing genes. In this context, Nrf1b may function as an activator of oxidative stress response genes. Although these findings suggest that the repertoire of target genes regulated by Nrf1b overlaps with Nrf2, further experiments are required to identify Nrf1b target genes in vivo.

In conclusion, we have identified an alternative Nrf1 transcript that encodes the Nrf1b protein that may play an important role in oxidative stress response. The regulation, function and implications of Nrf1b isoform expression for Nrf1-dependent gene expression and cellular homeostasis in various cell types in vivo remain to be explored.

## References

[1] Andrews NC, Erdjument-Bromage H, Davidson MB, Tempst P, Orkin SH (1993) Erythroid transcription factor NF-E2 is a haematopoietic-specific basic-leucine zipper protein. Nature 362: 722–728. 8469283 10.1038/362722a0

[2] Chan JY, Han XL, Kan YW (1993) Cloning of Nrf1, an NF-E2-related transcription factor, by genetic selection in yeast. Proc Natl Acad Sci U S A 90: 11371–11375. 8248256 10.1073/pnas.90.23.11371 PMC47984

[3] Kobayashi A, Ito E, Toki T, Kogame K, Takahashi S, et al (1999) Molecular cloning and functional characterization of a new Cap‘n’ collar family transcription factor Nrf3. J Biol Chem 274: 6443–6452. 10037736 10.1074/jbc.274.10.6443

[4] Moi P, Chan K, Asunis I, Cao A, Kan YW (1994) Isolation of NF-E2-related factor 2 (Nrf2), a NF-E2-like basic leucine zipper transcriptional activator that binds to the tandem NF-E2/AP1 repeat of the beta-globin locus control region. Proc Natl Acad Sci U S A 91: 9926–9930. 7937919 10.1073/pnas.91.21.9926 PMC44930

[5] Motohashi H, O'Connor T, Katsuoka F, Engel JD, Yamamoto M (2002) Integration and diversity of the regulatory network composed of Maf and CNC families of transcription factors. Gene 294: 1–12. 12234662 10.1016/s0378-1119(02)00788-6

[6] Chan JY, Kwong M, Lu R, Chang J, Wang B, et al (1998) Targeted disruption of the ubiquitous CNC-bZIP transcription factor, Nrf-1, results in anemia and embryonic lethality in mice. Embo J 17: 1779–1787. 9501099 10.1093/emboj/17.6.1779 PMC1170525

[7] Johnsen O, Murphy P, Prydz H, Kolsto AB (1998) Interaction of the CNC-bZIP factor TCF11/LCR-F1/Nrf1 with MafG: binding-site selection and regulation of transcription. Nucleic Acids Res 26: 512–520. 9421508 10.1093/nar/26.2.512 PMC147270

[8] Nguyen T, Sherratt PJ, Pickett CB (2003) Regulatory mechanisms controlling gene expression mediated by the antioxidant response element. Annual review of pharmacology and toxicology 43: 233–260. 10.1146/annurev.pharmtox.43.100901.140229 12359864

[9] Kwak MK, Wakabayashi N, Greenlaw JL, Yamamoto M, Kensler TW (2003) Antioxidants enhance mammalian proteasome expression through the Keap1-Nrf2 signaling pathway. Molecular and cellular biology 23: 8786–8794. 14612418 10.1128/MCB.23.23.8786-8794.2003 PMC262680

[10] Kensler TW, Wakabayashi N, Biswal S (2007) Cell survival responses to environmental stresses via the Keap1-Nrf2-ARE pathway. Annual review of pharmacology and toxicology 47: 89–116. 10.1146/annurev.pharmtox.46.120604.141046 16968214

[11] Li J, Lee JM, Johnson JA (2002) Microarray analysis reveals an antioxidant responsive element-driven gene set involved in conferring protection from an oxidative stress-induced apoptosis in IMR-32 cells. The Journal of biological chemistry 277: 388–394. 11687587 10.1074/jbc.M109380200

[12] Xu Z, Chen L, Leung L, Yen TS, Lee C, et al (2005) Liver-specific inactivation of the Nrf1 gene in adult mouse leads to nonalcoholic steatohepatitis and hepatic neoplasia. Proc Natl Acad Sci U S A 102: 4120–4125. 15738389 10.1073/pnas.0500660102 PMC554825

[13] Lee CS, Lee C, Hu T, Nguyen JM, Zhang J, et al (2011) Loss of nuclear factor E2-related factor 1 in the brain leads to dysregulation of proteasome gene expression and neurodegeneration. Proceedings of the National Academy of Sciences of the United States of America 108: 8408–8413. 21536885 10.1073/pnas.1019209108 PMC3100960

[14] Ohtsuji M, Katsuoka F, Kobayashi A, Aburatani H, Hayes JD, et al (2008) Nrf1 and Nrf2 play distinct roles in activation of antioxidant response element-dependent genes. J Biol Chem 283: 33554–33562. 18826952 10.1074/jbc.M804597200 PMC2662273

[15] Radhakrishnan SK, Lee CS, Young P, Beskow A, Chan JY, et al (2010) Transcription factor Nrf1 mediates the proteasome recovery pathway after proteasome inhibition in mammalian cells. Mol Cell 38: 17–28. 20385086 10.1016/j.molcel.2010.02.029 PMC2874685

[16] Xing W, Singgih A, Kapoor A, Alarcon CM, Baylink DJ, et al (2007) Nuclear factor-E2-related factor-1 mediates ascorbic acid induction of osterix expression via interaction with antioxidant-responsive element in bone cells. J Biol Chem 282: 22052–22061. 17510056 10.1074/jbc.M702614200

[17] Berg DT, Gupta A, Richardson MA, O'Brien LA, Calnek D, et al (2007) Negative regulation of inducible nitric-oxide synthase expression mediated through transforming growth factor-beta-dependent modulation of transcription factor TCF11. J Biol Chem 282: 36837–36844. 17928287 10.1074/jbc.M706909200

[18] Narayanan K, Ramachandran A, Peterson MC, Hao J, Kolsto AB, et al (2004) The CCAAT enhancer-binding protein (C/EBP)beta and Nrf1 interact to regulate dentin sialophosphoprotein (DSPP) gene expression during odontoblast differentiation. J Biol Chem 279: 45423–45432. 15308669 10.1074/jbc.M405031200

[19] Husberg C, Murphy P, Bjorgo E, Kalland KH, Kolsto AB (2003) Cellular localisation and nuclear export of the human bZIP transcription factor TCF11. Biochimica et biophysica acta 1640: 143–151. 12729924 10.1016/s0167-4889(03)00041-7

[20] Husberg C, Murphy P, Martin E, Kolsto AB (2001) Two domains of the human bZIP transcription factor TCF11 are necessary for transactivation. J Biol Chem 276: 17641–17652. 11278371 10.1074/jbc.M007951200

[21] Wang W, Kwok AM, Chan JY (2007) The p65 isoform of Nrf1 is a dominant negative inhibitor of ARE-mediated transcription. J Biol Chem 282: 24670–24678. 17609210 10.1074/jbc.M700159200

[22] Wang W, Chan JY (2006) Nrf1 is targeted to the endoplasmic reticulum membrane by an N-terminal transmembrane domain. Inhibition of nuclear translocation and transacting function. J Biol Chem 281: 19676–19687. 16687406 10.1074/jbc.M602802200

[23] Zhang Y, Crouch DH, Yamamoto M, Hayes JD (2006) Negative regulation of the Nrf1 transcription factor by its N-terminal domain is independent of Keap1: Nrf1, but not Nrf2, is targeted to the endoplasmic reticulum. Biochem J 399: 373–385. 16872277 10.1042/BJ20060725 PMC1615900

[24] Steffen J, Seeger M, Koch A, Kruger E (2010) Proteasomal degradation is transcriptionally controlled by TCF11 via an ERAD-dependent feedback loop. Mol Cell 40: 147–158. 20932482 10.1016/j.molcel.2010.09.012

[25] Zhang Y, Lucocq JM, Yamamoto M, Hayes JD (2007) The NHB1 (N-terminal homology box 1) sequence in transcription factor Nrf1 is required to anchor it to the endoplasmic reticulum and also to enable its asparagine-glycosylation. Biochem J 408: 161–172. 17705787 10.1042/BJ20070761 PMC2267355

[26] Zhao R, Hou Y, Xue P, Woods CG, Fu J, et al (2011) Long isoforms of NRF1 contribute to arsenic-induced antioxidant response in human keratinocytes. Environ Health Perspect 119: 56–62. 20805060 10.1289/ehp.1002304 PMC3018500

[27] Biswas M, Phan D, Watanabe M, Chan JY (2011) The Fbw7 tumor suppressor regulates nuclear factor E2-related factor 1 transcription factor turnover through proteasome-mediated proteolysis. The Journal of biological chemistry 286: 39282–39289. 21953459 10.1074/jbc.M111.253807 PMC3234752

[28] Tsuchiya Y, Morita T, Kim M, Iemura S, Natsume T, et al (2011) Dual regulation of the transcriptional activity of Nrf1 by beta-TrCP- and Hrd1-dependent degradation mechanisms. Molecular and cellular biology 31: 4500–4512. 21911472 10.1128/MCB.05663-11 PMC3209242

[29] Mironov AA, Fickett JW, Gelfand MS (1999) Frequent alternative splicing of human genes. Genome research 9: 1288–1293. 10613851 10.1101/gr.9.12.1288 PMC310997

[30] Ayoubi TA, Van De Ven WJ (1996) Regulation of gene expression by alternative promoters. FASEB journal : official publication of the Federation of American Societies for Experimental Biology 10: 453–460. 8647344

[31] Baek D, Davis C, Ewing B, Gordon D, Green P (2007) Characterization and predictive discovery of evolutionarily conserved mammalian alternative promoters. Genome research 17: 145–155. 17210929 10.1101/gr.5872707 PMC1781346

[32] Gascard P, Parra MK, Zhao Z, Calinisan VR, Nunomura W, et al (2004) Putative tumor suppressor protein 4.1B is differentially expressed in kidney and brain via alternative promoters and 5′ alternative splicing. Biochimica et biophysica acta 1680: 71–82. 15488987 10.1016/j.bbaexp.2004.08.006

[33] Cianetti L, Di Cristofaro A, Zappavigna V, Bottero L, Boccoli G, et al (1990) Molecular mechanisms underlying the expression of the human HOX-5.1 gene. Nucleic acids research 18: 4361–4368. 1975093 10.1093/nar/18.15.4361 PMC331252

[34] Azizkhan JC, Jensen DE, Pierce AJ, Wade M (1993) Transcription from TATA-less promoters: dihydrofolate reductase as a model. Critical reviews in eukaryotic gene expression 3: 229–254. 8286846

[35] Kwak MK, Itoh K, Yamamoto M, Kensler TW (2002) Enhanced expression of the transcription factor Nrf2 by cancer chemopreventive agents: role of antioxidant response element-like sequences in the nrf2 promoter. Molecular and cellular biology 22: 2883–2892. 11940647 10.1128/MCB.22.9.2883-2892.2002 PMC133753

[36] Sandelin A, Carninci P, Lenhard B, Ponjavic J, Hayashizaki Y, et al (2007) Mammalian RNA polymerase II core promoters: insights from genome-wide studies. Nature reviews Genetics 8: 424–436. 10.1038/nrg2026 17486122

[37] Mulac-Jericevic B, Conneely OM (2004) Reproductive tissue selective actions of progesterone receptors. Reproduction 128: 139–146. 15280552 10.1530/rep.1.00189

